# *CCP*4*i*2: the new graphical user interface to the *CCP*4 program suite

**DOI:** 10.1107/S2059798317016035

**Published:** 2018-02-01

**Authors:** Liz Potterton, Jon Agirre, Charles Ballard, Kevin Cowtan, Eleanor Dodson, Phil R. Evans, Huw T. Jenkins, Ronan Keegan, Eugene Krissinel, Kyle Stevenson, Andrey Lebedev, Stuart J. McNicholas, Robert A. Nicholls, Martin Noble, Navraj S. Pannu, Christian Roth, George Sheldrick, Pavol Skubak, Johan Turkenburg, Ville Uski, Frank von Delft, David Waterman, Keith Wilson, Martyn Winn, Marcin Wojdyr

**Affiliations:** a York Structural Biology Laboratory, Department of Chemistry, University of York, York YO10 5DD, England; b STFC Rutherford Appleton Laboratory, Chilton, Didcot OX11 0QX, England; c MRC Laboratory of Molecular Biology, Francis Crick Avenue, Cambridge CB2 0QH, England; d University of Newcastle upon Tyne, Northern Institute for Cancer Research, Framlington Place, Newcastle upon Tyne NE2 4HH, England; e Biophysical Structural Chemistry, Leiden University, PO Box 9502, 2300 RA Leiden, The Netherlands; f Department of Structural Chemistry, Georg-August-Universität Göttingen, Tammannstrasse 4, 37077 Göttingen, Germany; g Nuffield Department of Medicine, University of Oxford, Oxford OX3 7BN, England; h Diamond Light Source Ltd, Harwell Science and Innovation Campus, Didcot OX11 0QX, England

**Keywords:** graphical user interfaces, structure solution, *CCP*4, *CCP*4*i*2, automation, pipelines

## Abstract

The *CCP*4 (Collaborative Computational Project, Number 4) software suite for macromolecular structure determination by X-ray crystallography groups brings together many programs and libraries that, by means of well established conventions, interoperate effectively without adhering to strict design guidelines. Because of this inherent flexibility, users are often presented with diverse, even divergent, choices for solving every type of problem. Recently, CCP4 introduced *CCP*4*i*2, a modern graphical interface designed to help structural biologists to navigate the process of structure determination, with an emphasis on pipelining and the streamlined presentation of results. In addition, *CCP*4*i*2 provides a framework for writing structure-solution scripts that can be built up incrementally to create increasingly automatic procedures.

## Abstract 2

*CCP*4*i*2 is a graphical user interface to the *CCP*4 (Collaborative Computational Project, Number 4) software suite and a Python language framework for software automation.

## 1. Introduction   

CCP4 (Collaborative Computational Project, Number 4) began in 1979 as a forum for collaboration between academic macromolecular crystallography (MX) software developers. Today, it is best known for supporting and releasing a suite of programs (Winn *et al.*, 2011 ▸) that have been contributed by a wide range of developers, and for organizing meetings and workshops that are particularly geared to educating inexperienced crystallographers. CCP4 has several permanent staff who ensure that the software suite is robust, multi-platform, easy to install, regularly updated and well documented. They provide online support and training workshops, and maintain an active peer-supported bulletin board.

Prior to about 2003, crystallographers using the program suite normally had to use the command line and scripting to run the programs. *CCP*4*i* (Potterton *et al.*, 2002 ▸), the first widely used graphical user interface (GUI) to the suite, simplified and expedited use of the suite and provided tools to view files and to track the structure-solution process in a database. Similar graphical interfaces for MX programs include *HKL*2*MAP* (Pape & Schneider, 2004 ▸), *HKL*-3000 (Minor *et al.*, 2006 ▸) and *PHENIX* (Echols *et al.*, 2012 ▸). A different approach, which provides a graphical view of electron-density maps and models with interactive tools geared mostly to model building, is used in *Coot* (Emsley *et al.*, 2010 ▸).

The graphical interface in *CCP*4*i*, based on the poorly supported Tcl/Tk toolkit, is now out of date, and consequently the time is right for the *CCP*4*i*2 project to provide an alternative automation and GUI system, informed by more modern GUI principles and the lessons learned from the implementation of *CCP*4*i*. The key objectives are to provide high-level tasks that automate the main stages of structure solution and to remove the need for specialized knowledge of file formats, program input, program log-file organization and so forth. Within *CCP*4*i*2 the workflow and the scientific decisions that control the tasks and display the task results are designed to be as transparent as possible, and care has been taken to keep the underlying data accessible. A key objective for *CCP*4*i*2 is to improve accessibility for inexperienced crystallographers, enabling straightforward structure solution using a default approach, with clear reports and documentation allowing users to understand the process and investigate results. We have also sought to enable more experienced crystallographers to fine-tune each step to address more difficult problems. Key information about the structure-solution process is captured in a database, which maintains a record of all of the jobs run and all of the data imported and generated.

### 1.1. The user interface   

**Figure 1:**
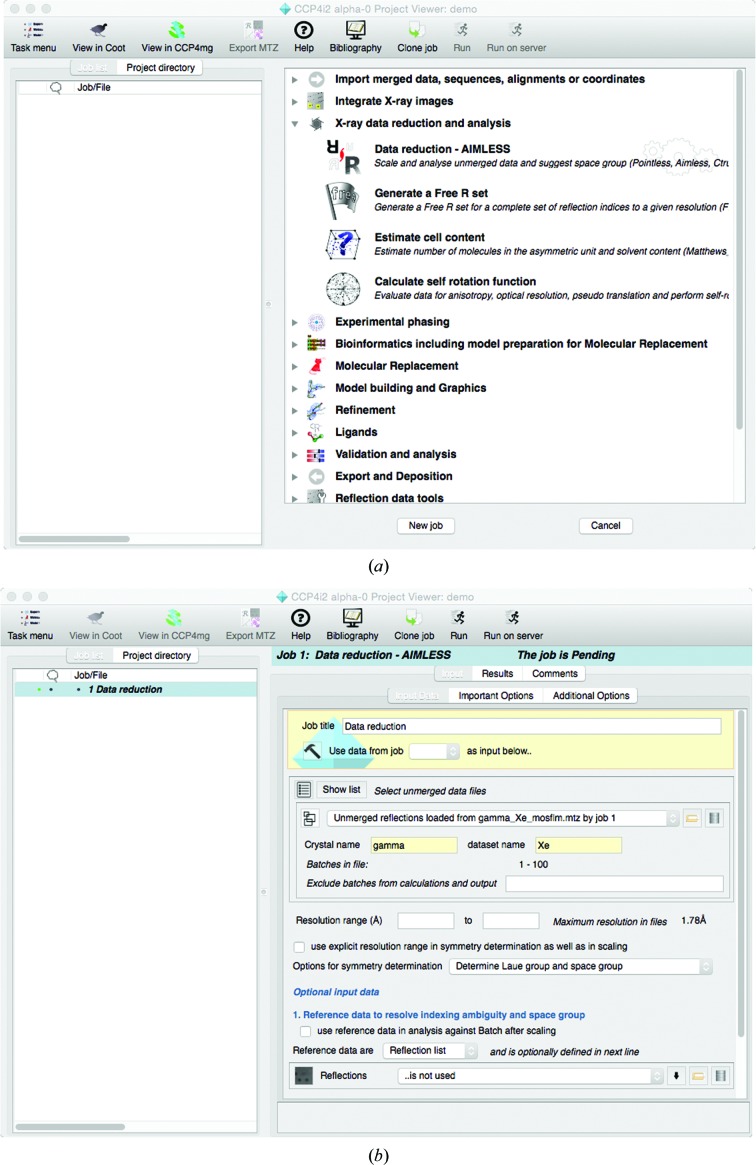

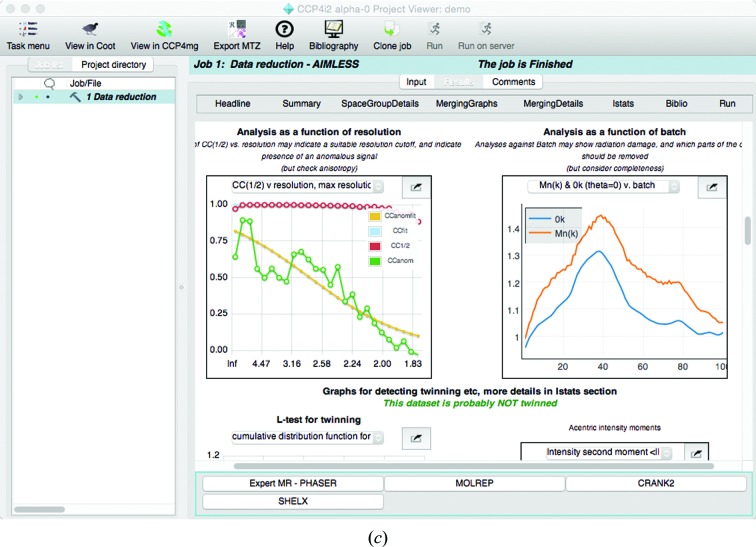
Three views of the project window. (*a*) The task menu. (*b*) A task input frame. (*c*) A task report.

*CCP*4*i*2 comprises three main elements: a database-backed project/job-management system, a scripting/reporting framework and a graphical user interface to these elements. The majority of a user’s experience of a software suite is determined by its user interface, and this is described here. The user is encouraged to organize their work into projects: typically, one project might result in one solved structure. The user views the status of a project through a project window, as shown in Fig. 1 ▸. The left-hand side of this window is the ‘job list’, a nested list of all jobs and data objects associated with the project, and the right-hand side is initially a ‘task menu’, a list of all available tasks organized into modules corresponding to the stages in structure solution. When the user chooses a task from the menu, the menu is replaced by the appropriate ‘task input’ frame. When a job has been executed, the ‘task input’ frame is replaced by a ‘report’ frame which presents the results from the task. For longer running tasks the ‘report’ frame will be updated continuously while the task is running.

**Figure 2:**
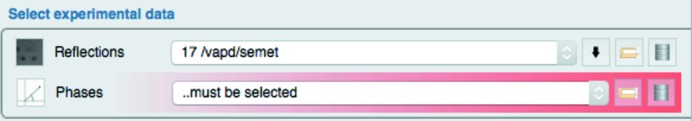
Fragment of a typical task input showing the widgets to select data corresponding to ‘Reflections’ and ‘Phases’.

The ‘Input’ usually has several tabs, with the topmost tab being labelled ‘input data’ and allowing the user to select the input data for the task. The other tabs provide control over different aspects of the task, but typically the user only needs to select sufficient input data for the task, which will then run with sensible default parameters. All data objects (coordinates, reflections, phases *etc.*) are stored in discrete files, but the database maintains a user-editable label for each file, which is intended to be more meaningful than the file’s path in the filesystem. The crystallographer using *CCP*4*i*2 is encouraged to think in terms of a ‘data object’ rather than a ‘file’: the user need only be concerned with the data content, as the software will handle file storage in the background. The widgets to select input data all have a similar appearance (Fig. 2 ▸), with a drop-down menu that lists the labels of any appropriate data that are already present in the project. Such data must have been either imported or created by a previous job in the project. In many situations defaults are selected automatically, being the data from the most recently run job. There are buttons to the right of the drop-down menu to download data from web servers, to select data from other projects in the *CCP*4*i*2 database or to import files from the local filesystem. On the left of the widget is the ‘icon button’, with an icon indicating the type of data and an associated contextual menu to access documentation as well as functionality to display, copy or paste data. The contextual menu offers additional functionality specific to the given data type, such as selecting a limited set of atoms from a coordinate data object.

Tasks are designed to require minimal user input. Where input is essential but not yet provided by the user, the corresponding widget is highlighted in red. Similarly, control parameters provided by the task interface are validated against developer-specified criteria, with inappropriate input highlighted in red and execution of the task prevented until inconsistencies have been addressed. The interface is dynamic in that the available detailed options may be updated dependent on the user’s data selection.

All files and jobs have a label assigned by default. However, the user may edit these labels to make them more meaningful and descriptive, which may aid them when reviewing the project in the future. There is also a ‘Comment’ tab next to the ‘Input’ and ‘Results’ tabs where the user may enter more detailed comments on a specific job.

**Figure 3:**
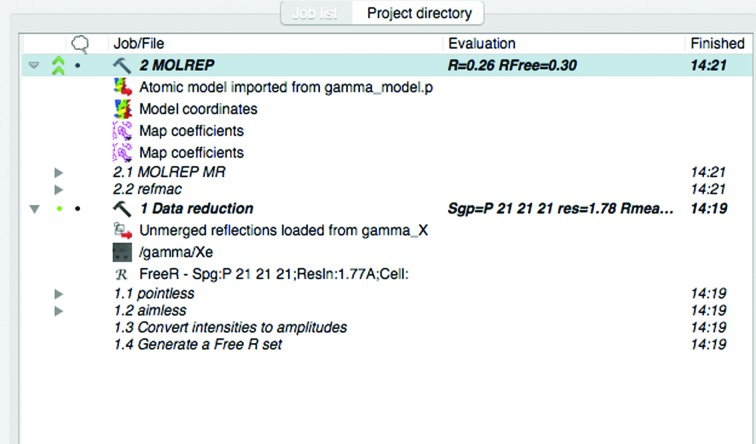
A job list showing that two jobs have been run in the project (‘Data reduction’ and ‘*MOLREP*’) and the sub-jobs and files associated with these jobs.

The job list (Fig. 3 ▸) shows all of the jobs associated with the project, listed with the most recent first and with icons indicating status (for example ‘pending’ or ‘running’). Many jobs have an associated ‘evaluation’, for example the *R* values for refinement. There is also a column for icons indicating the user’s manual evaluation such as ‘best’, ‘good’ or ‘rejected’. Jobs are presented hierarchically in a tree-view widget: the disclosure triangle of each job can be clicked to reveal ‘sub-jobs’ spawned by the parent job, and data objects imported or generated thereby. Each entry in the tree view (job, sub-job or data object) has an associated contextual menu which provides access to relevant functionality. For jobs and sub-jobs this functionality may be, for example, viewing associated log files, while for data objects it may enable opening in a suitable viewing utility. Thus, the user can drill down to see the details of a particular job. *CCP*4*i*2 makes use of other graphical viewers to display the contents of files. The *CCP*4 program *qtRView* displays program log files. *ViewHKL* (Krissinel & Evans, 2012 ▸) is used to view MTZ experimental data files. *Coot* (Emsley *et al.*, 2010 ▸) and *CCP*4*mg* (McNicholas *et al.*, 2011 ▸) are used to view maps and coordinate files. A ‘project directory’ tab next to the ‘job list’ tab displays the contents of the file-system directory in which the job was executed, providing access to information that may be of interest to software developers or expert users.

Extensive documentation for *CCP*4*i*2 users and developers is provided with the program and on the CCP4 website. The documentation includes an introductory ‘Quickstart’ tutorial (also available as a YouTube video at https://www.youtube.com/watch?v=fB7BRVzBURg), discussion of the different types of data used in *CCP*4*i*2 and comprehensive documentation on all of the tasks, explaining both the task-input options and the reports.

## 2. Overview of implementation   

*CCP*4*i*2 is written in the Python scripting language (http://www.python.org). This was chosen, at least partly, to enable easier collaboration with other major macromolecular crystallography packages. The huge variety of libraries both bundled with Python and available as easily installable third-party add-ons has made it an attractive choice for many projects. The graphical toolkit used in *CCP*4*i*2 is Qt (https://www.qt.io/), which has a Python interface provided by the PyQt project (https://riverbankcomputing.com/software/pyqt/intro). Several other Python language software tools are used as listed in Table 1 ▸. The SWIG system (http://www.swig.org) has been used to provide a Python interface to scientific C++ libraries used in *CCP*4, including the model coordinate library MMDB (Krissinel *et al.*, 2004 ▸) and the crystallographic library Clipper (Cowtan, 2003 ▸). All of these tools are collected together as a bespoke Python distribution, termed ccp4-python, which is distributed as part of the *CCP*4 suite. ccp4-python is available for the three most widely used desktop platforms (Windows, MacOSX and Linux) and provides an excellent range of tools that allow developers to distribute software for which the *CCP*4 distribution provides all essential dependencies. Both the graphical interface and the scientific scripts are written in Python so they both have access to ccp4-python and the data model and the range of scientific functionality that are encoded in *CCP*4*i*2.

The *CCP*4*i*2 software comprises two components: the ‘core’ software which provides the framework including the graphical interface, database and data model, and the ‘tasks’ that encode the scientific functionality and usually provide a ‘wrapper’ around the computational crystallography programs included in the *CCP*4 suite. Each wrapper generates the appropriate inputs for the wrapped program, runs the program and handles the program outputs. Some crystallo­graphic software in the suite is directly accessible *via* a Python interface so that the ‘wrapper’ script can use this interface rather than wrapping and executing a separate program. The programming interface to a wrapper script is a set of Python functions with strictly defined interfaces, which means that ‘pipeline’ scripts can straightforwardly be generated by combining multiple wrapper scripts and so running multiple different programs. Within *CCP*4*i*2 both wrappers and pipelines are referred to as ‘tasks’. The consistent Python interface to the tasks also aids in creating consistent graphical interfaces with minimal programming effort. The tasks deal with idiosyncrasies of the individual programs and also encode a large amount of crystallographic expertise to promote the optimal use of the *CCP*4 software by inexpert crystallographers. The fundamental idea of providing a consistent Python interface to ‘tasks’ is in common with other structure-solution systems such as *PHENIX* and *xia*2, and makes interfacing to software developed in these systems feasible.

The tasks have been implemented by many different developers with expertise in different aspects of structure solution, covering the complete structure-solution process from processing data to validation and analysis of the final structure. Although most tasks are running nongraphical programs, there are certain tasks that run interactive software such as *Coot* and *CCP*4*mg*. The integration of these programs into the *CCP*4*i*2 framework makes their use less error-prone, simplifies movement of data between applications, and creates a permanent record of the data and files that are used and created during that process.

The key advance in *CCP*4*i*2 over *CCP*4*i* (and the similar *PHENIX* GUI) is the clear tracking of all data in a project. This not only improves the user interface, but importantly also serves as a long-term record of the structure-solution process. Data tracking is made possible by implementing a data model, a clear definition of all of the types of data used in each step of the process, and by maintaining a robust database. In the data model each data type is represented by a Python class. The classes cover a range of complexity, for example CInt, an integer; CCell, crystallographic unit-cell parameters; CMtzDataFile, a reference to an MTZ data file; and CEnsemble, a full description of an ensemble of models for input to molecular replacement. The Python data classes provide many utility functions. For example, CMtzDataFile has functions to return information from the MTZ file. For each data class there is an appropriate graphical widget that is used in the interface. All tasks have input and output data clearly defined in terms of the Python data classes so that data can be passed seamlessly between tasks. The input and output data are saved in conventional file formats [for example PDB (Callaway *et al.*, 1996 ▸) or CIF (Westbrook & Fitzgerald, 2009 ▸) for model coordinates, MTZ for reflection data] and internally the *CCP*4*i*2 data class only keeps track of the name of the file and not the actual scientific data.

The database keeps a record of all jobs run and all files used. The key data in the *CCP*4*i*2 database are ‘projects’, ‘jobs’, ‘files’ and ‘file uses’. A job corresponds to an instance in which a *CCP*4*i*2 task is run. Each job is associated with one project. For each job all of the output data (a ‘file’ in the database) and input data (a ‘file use’ in the database) are recorded.

Each *CCP*4*i*2 project has an associated directory structure in which all files associated with the project are saved in a strictly organized fashion. A copy of any file imported into the project is always saved in the project directory and all files associated with any given job are automatically saved in a subdirectory for that job.

### 2.1. The data model   

*CCP*4*i*2 has clearly defined data types, and all data and parameters in the interface and scripts must be of one of the defined types. This approach enables easier transfer of data between different tasks and between the graphical interface, scripts and database. Each data type is represented by a Python class that provides relevant functionality, and each data type has an associated graphical widget so that the user sees a consistent representation.

*CCP*4*i*2, as far as possible, guides the user to input appropriate parameters and warns if inputs are invalid or missing. Task developers can associate criteria with each control parameter of a task. Examples of such criteria include a minimum allowed value, a maximum allowed value, a default value and whether the value can be left undefined. These criteria are stored in the qualifiers property of the *CCP*4*i*2 data classes and can be set for each instance of the class representing one task parameter. Most qualifiers are relevant for data validation or representation in the user interface. An example of the former: a CInt (integer) can have specified max and/or min qualifiers which define an allowed range for the integer. Examples of the latter are the guiLabel and toolTip qualifiers that specify the default label and ‘pop-up’ help for the parameter.

**Figure 4:**
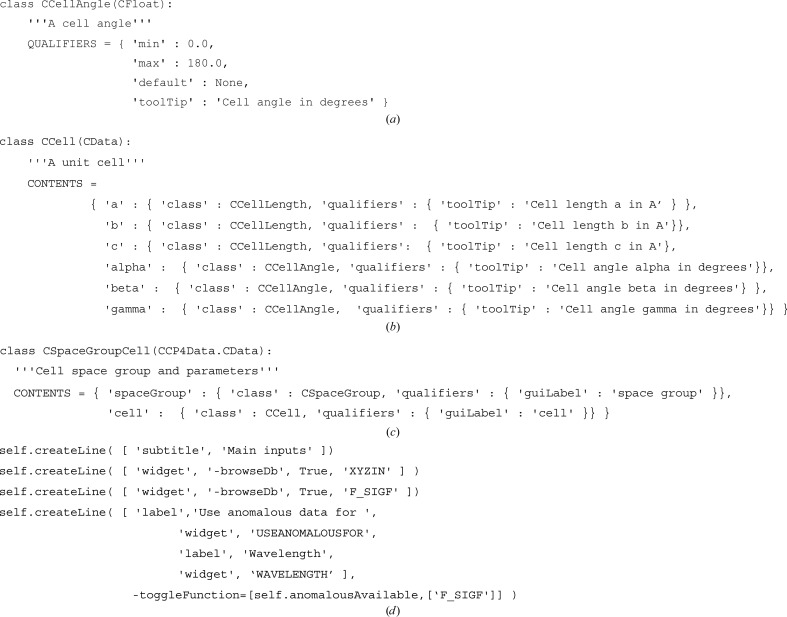
Examples of code. (*a*) Definition for cell angles. (*b*) Definition of a class to handle cell parameters. (*c*) The CSpaceGroupCell class. (*d*) Task input for refinement using *REFMAC*5.

The basic data classes are CInt (an integer), CFloat (a floating point number), CBoolean (a Boolean), CString (a string) and CList (a list). More specific data classes can be subclassed from these; for example, the definition for cell angles (Fig. 4 ▸ *a*).

Here, CCellAngle is derived from CFloat with max and min qualifiers set so that the validity checking in the CFloat.validity() method will flag an error for a value outside the allowed range of 0–180°. The default is None since there is no reasonable ‘best guess’ value and the toolTip which will appear on the user interface reminds the user that the value is in degrees. The only additional code for the class are the methods getRadians() and setRadians(), which enable the input and output of a value in radians.

More complex data can be composed from multiple basic data classes; for example, all that is necessary to define a class to handle cell parameters (Fig. 4 ▸ *b*).

Here, CCell is derived from CData, which is the base class for complex data and provides generic functionality and CONTENTS is a dictionary specifying the cell parameters *a*, *b*, *c*, α, β and γ. Each of these components has a class specified, and the toolTip qualifier is also redefined to inform the user which component in the cell it is. No more code is necessary to define the CCell class; when it is instantiated the CData.build() method builds the data structures based on the CONTENTS definition and all essential functionality is inherited from CData.

There is also a CSpaceGroup class which is derived from CString but has an important validity() method to check that the space group is valid and a fix() method which, amongst other things, will ‘fix’ a value input in an alternative space-group convention by converting it to the Hermann–Mauguin convention. The next level of complexity is the CSpaceGroupCell class, which is composed from CCell and CSpaceGroup (Fig. 4 ▸ *c*).

The CSpaceGroupCell.validity() method first calls CCell.validity() and CSpaceGroup.validity() to ensure that the components are valid and then checks that the cell parameters are appropriate for the space group.

Classes to handle all data used within *CCP*4*i*2 are built up following similar principles to the cell and space-group examples. For each data class in *CCP*4*i*2 there is a graphical widget to represent the data in the graphical user interface.

The most important classes are those that handle data files. All data used in *CCP*4*i*2 are saved in files which are usually in the conventional formats such as PDB or mmCIF for model coordinates and MTZ for experimental data. The CDataFile class handles the reference to the file and has subclasses such as CPdbDataFile for model data and CMtzDataFile for experimental data. Use of a particular data-file class indicates that the corresponding data object is of a particular type, but the file-handling classes also have concepts of file ‘subtype’ and ‘file contents’ which can give more information such as whether reflection data are in the form of structure-factor amplitudes or intensities, and whether a coordinate file contains a full model, a fragment of the structure, heavy atoms or a homologue. Although these categories cannot always be clearly defined, they can be useful in guiding the selection of appropriate files for a particular task. When the data file is recorded in the database, its filetype and the subtype and file content are also recorded. Thus, the descendants of the CDataFile have properties that define those metadata of the file that are relevant to its use in *CCP*4*i*2.

CDataFile classes provide an application programming interface (API) to access the actual data in the file. The accessible data are limited to those which have been found to be useful for the *CCP*4*i*2 interface or scripts. Access to the files is often *via* Python interfaces to the usual CCP4 C++ libraries such as MMDB for coordinate files and Clipper for MTZ files.

A major change in *CCP*4*i*2 is in the way that experimental data are handled. The MTZ file format is designed to hold all possible types of experimental data (such as structure-factor amplitudes, phases and free *R* flags) with one set of data per column in the file. Multiple columns are needed for some data, for example intensities, and their errors comprise two columns. Most programs in the *CCP*4 suite expect only one input MTZ file and will output one MTZ file that is a copy of the input file with new data appended in additional columns. To use these programs through older interfaces such as *CCP*4*i*, it is necessary for the user to select an input MTZ file and then specify which columns from the file are to be used. This was a two-step process, which has now been simplified to one step in *CCP*4*i*2 by organizing the data within a separate ‘mini-MTZ’ for each self-sufficient set of data. The different mini-MTZs contain between one and four columns of data. There are four types of mini-MTZ.

(i) Reflection data: the merged structure-factor amplitudes or intensities, either in anomalous pairs of reflections or mean values.
(ii) Phase probability distributions, represented either as a phase with an associated figure of merit (FOM) or as Hendrickson–Lattmann coefficients.
(iii) Map coefficients, corresponding to a weighted structure-factor amplitude and associated phase.
(iv) Free *R* flags.

Both reflection data and phases have alternative representations which are interconvertible, although some representations carry more information and thus are preferred. *CCP*4*i*2 has tools to interconvert representations and will do so automatically for input to any program that can only handle a particular representation. Since most programs still expect only one MTZ input file and output only one MTZ file, the *CCP*4*i*2 wrapper scripts merge the mini-MTZs into one input file before invoking an underlying program, and extract the useful new data from the output MTZ file when the program has finished. Although traditional MTZ files are still created by various *CCP*4 programs ‘under the hood’ and are converted to and from mini-MTZ files when required, the user should only see the widgets that correspond to data types in *CCP*4*i*2 and need only be exposed to the traditional many-column MTZ file when importing an MTZ file from outside *CCP*4*i*2 or when exporting data, for example at the end of the project for deposition. Using mini-MTZs rather than traditional MTZ files that can have large sets of redundant data should help to reduce disk-space requirements when using *CCP*4*i*2, but this saving is also dependent on a cleanup mechanism that removes redundant working files (often large MTZ files) and intermediate files from pipelines with multiple steps.

### 2.2. Database   

All of the key scientific data for a project are saved in files in the project directory, but a relational database is used to keep a record of these files and their provenance. A full record of a project comprises the project directory and the database contents specific to the project, and this full record can be exported and imported as a single bundle, as described below.

The current implementation of the database is based on SQLite v.3 (https://www.sqlite.org/) accessed *via* the Python sqlite3 interface (https://docs.python.org/2/library/sqlite3.html). The key advantage compared with most other relational database-management systems is the licensing, which permits free distribution as part of the *CCP*4 suite. SQLite is not a client–server system but is embedded in the *CCP*4*i*2 software. This means that each database file is effectively only accessible to one *CCP*4*i*2 user. In the future, we anticipate that *CCP*4*i*2 will support a multi-user client–server database; the *CCP*4*i*2 database interface has been written with this in mind and should be easily portable.

Some of the key data tables in the database are listed in Table 2 ▸. Each item in each table has an automatically assigned UUID (Universally Unique IDentifier, http://pubs.opengroup.org/onlinepubs/9629399/apdxa.htm), which is used for cross-referencing with items in other tables.

Each project is associated with one user and has a user-given name and recorded project directory. In the database schema a project has one owner and other users can have varying levels of access. This multi-user access is not available in the present SQLite implementation, but it will be useful when a client–server system is implemented. Projects can be organized into a hierarchy, and to support this property each project may have a parent project.

All jobs are associated with a project and are recorded with the task name, the current job status (*e.g.* ‘running’ or ‘finished’) and an editable title. Pipeline tasks with the corresponding subtasks are recorded in the database with the parent job identifier (the parent’s UUID).

A record for each file that is imported or created in the project is entered into the database. The record contains the file’s path, type, any user annotation and content (as explained above). Whenever a file is used as input to a job, it is recorded in the ‘file use’ table. Besides the annotations of projects, jobs and files that are saved in the database, for many tasks key progress parameters such as ‘percentage built’ or ‘*R* factor’ are recorded in the database. These parameters can then be rapidly retrieved and displayed in the job list as a quick reference for users.

All access to the database is *via* a Python API, the CDbApi class, which provides tools to create, modify, delete and query items in each table in the database. There are also more customized tools for frequently used functions, particularly for the common queries needed to support the graphical user interface. The database is important in controlling the running of a job and for communication between the graphical front-end process and jobs running in background processes. When the user creates a new job in the interface, a job is recorded in the database with status ‘pending’. When the user runs the job the parameters set in the interface are written to a file named input_params.xml in the corresponding job directory and the job status is changed to ‘queued’. The job controller module, CJobController, polls the database for queued jobs and, provided that loading limitations are not reached, will start a new nongraphical process by running the runTask script with the input_params.xml file passed as an argument. The job status is then updated to ‘running’. The non­graphical process will update the database when the job finishes and will record the status as ‘finished’, ‘failed’ or ‘unsatisfactory’; the last of these statuses means that although there was no obvious failure, the task did not generate a useful result. If the running job is a pipeline with sub-jobs then the sub-jobs and their output files are recorded in the database. When a job finishes, the job parameters are written to a params.xml file; this is usually very similar to the input_params.xml file but has the corresponding ‘output data’ section populated. The contents of params.xml is also passed to the database API CDbApi.gleanJobFiles(), which scans for output file and job key data, which are loaded into the database. The input_params.xml and params.xml files serve as communication between the graphical interface, script and database, and remain in the project directory as a backup. This entire job-recording mechanism works without the implementers of individual tasks needing to access the database.

The graphical process polls the database for new jobs and changes in job status (entered by the nongraphical processes) and will update these in the job list so that the user can see progress; they can usually also see a report being updated in real time, but this is handled by a different mechanism.

As each job in the structure-solution process has an input_params.xml file which records the exact parameters used to run the job, these, along with the database record of the flow of data between jobs, provide all of the information needed to completely reproduce the structure solution.

### 2.3. The task application programming interface   

Many different developers have contributed tasks to the *CCP*4*i*2 project and it is therefore important that writing a task is straightforward. *CCP*4*i*2 provides a framework which performs as much of the generic work as possible, and the task implementation need only provide the fragments of functionality that must be customized for the task. Implementing a task normally requires the creation or tailoring from boilerplate code of four files.

(i) The def file is an XML file specifying all the input data, control parameters and output data for the task.
(ii) The script is a Python script which usually wraps a program or encodes a pipeline.
(iii) The GUI (graphical user interface) is a Python script defining the user interface.
(iv) The report file is a Python script defining the job report presented to the user after the job has finished (and in some cases while the job is running).

The def file is the definition of the interface to a task. The def file can be created using the provided graphical editor *defEd*. Whenever the *defEd* application is run it uses Python introspection tools to create a list of all the data classes within *CCP*4*i*2, their associated qualifiers and class documentation, so that the developer is presented with all available options. Alternatively, boilerplate code is provided together with tools to help to derive code for a new task. The def file is broadly equivalent to the Phil file used in the *PHENIX* software, and we are developing Phil-to-def file-conversion tools to simplify interfacing to software that already supports Phil files.

A task script is created by subclassing CPluginScript. Creating a program wrapper usually requires coding three methods.

(i) processInputFiles() is called before the program is run, and performs any input data conversion required by the program. A common requirement is merging the user-specified reflection-data objects into one MTZ file.
(ii) createCommandAndScript() is also called before the program is run and defines the command line and any input script for the program.
(iii) processOutputFiles() is called after the program has finished and performs any necessary file-format conversions to a *CCP*4*i*2 standard. It must also generate a program.xml file containing the data needed for the task report; if this is not provided by the program then the processOutputFile() method should provide logic to calculate such data or extract them from a log file.

Pipeline tasks are also derived from CPluginScript, but this requires reimplementing the process() method to control running a series of ‘subtasks’.

*CCP*4*i*2 can autogenerate a graphical interface for task inputs based on the list of parameters in the def file, but this is rarely ideal: a customized GUI script can organize parameters, provide helpful annotations and provide logic to deal with interdependent parameters, *i.e.* parameters whose relevance or optimal value depends in some way on the value of another parameter. Correct handling of interdependent parameters by the GUI script makes for a dynamic interface which customizes detailed options based on user selections and may ensure that the user is not presented with irrelevant options. *CCP*4*i*2 has a graphical widget class to represent each of the data classes and can therefore automatically insert the appropriate widget for each parameter specified in the task interface. The GUI script defines the graphical interface layout in terms of lines in the window using the createLine() method and through this can specify the widgets and labels to appear on a line.

**Figure 5:**
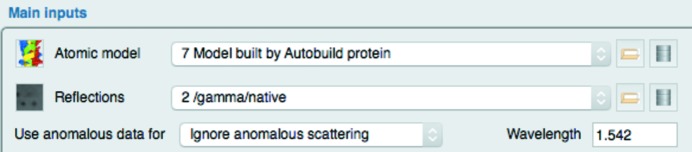
A fragment of the task input for the *REFMAC*5 task showing selection of ‘Atomic model’ and ‘Reflection’ data and a line of details for using anomalous data. This line is only shown if the user has selected Reflections that are anomalous data.

Fig. 5 ▸ shows a simple example from the interface to refinement using *REFMAC*5 (Murshudov *et al.*, 2011 ▸), where the user can select ‘Atomic model’ and ‘Reflections’ (parameter names in the code: XYZIN and F_SIGF) and then select how anomalous data are used (USEANOMALOUSFOR parameter) and enter the wavelength (WAVELENGTH parameter). If the user selects reflection data without anomalous data the final line is removed from the interface. This task input is encoded by the code in Fig. 4 ▸(*d*).

In this code each of four lines in the interface are specified by one call to createLine(); firstly specifying a ‘subtitle’ and then specifying a combination of ‘labels’ and ‘widgets’. The data type of the widget parameters has been specified in the def file, so the *CCP*4*i*2 framework is able to provide the correct widget. Some customizations of widgets are possible. For example, in this code the -browseDb argument is used to indicate drawing a ‘database’ icon in the widget through which the user can access all data in all projects. The final call to createLine() has an additional -toggleFunction argument that specifies a function, anomalousDataAvailable(), that will control the visibility of the fourth line. In this case, the implementation of anomalous­DataAvailable() returns True or False depending on the data available in the user’s selected input reflection-data object. The function is called automatically whenever the value of F_SIGF is changed by the user and will return a flag indicating whether the line should be displayed or not based on whether the user’s selected data file contains anomalous data.

The task-input interface is organized into tabs, with the first tab containing all the essential data selection and subsequent tabs containing less-used options.

*CCP*4*i*2 provides a report for all finished jobs. Additionally, for some tasks a short, frequently updated report is generated while the job is still running. The reports show detailed data from the job, usually presented as graphs and tables, including comments highlighting important aspects of the data. The data presented in the report comes from the program.xml file that is created either by the running the program or the task script. The report is an HTML file which is created in the *CCP*4*i*2 graphical process on demand if the user chooses to view a report that does not already exist.

The appearance of the HTML report file is defined by a Python-coded task-specific subclass of the Report class. Besides the Report class, there is a class for each of the report elements such as folders, graphs, tables, text and pictures. The task Report creates a hierarchy of these elements in an arrangement corresponding to the layout required in the HTML report. The Report class loads the data from the program.xml file into the appropriate report elements. After the report has been fully defined in this class instance, the Report.as_html() method is called; this returns an HTML file of the full report by calling all of the report elements to return an HTML representation of themselves.

A task-report class can include a definition for a ‘running’ report presented while the job is still running; typically, this will be a very short report such as a simple graph. The *CCP*4*i*2 graphical process updates the running report when it sees that the program.xml file created by the program has been updated.

The graph viewers developed for *CCP*4*i*2, *Pimple* and *JSPimple*, display graphs in the report page or can display graphs from log files. *JSPimple* is used in the *CCP*4*i*2 HTML report pages and is written in JavaScript using either the jquery.flot (http://www.flotcharts.org/) or the Plotly (https://plot.ly) backends. *Pimple* is a standalone application with additional graph editing, export and print functionality built using PyQt and the libraries Matplotlib (for graphs; https://matplotlib.org) and NumPy (for numerical calculations; http://www.numpy.org).

#### 2.3.1. Drop-in compatibility with *CCP*4 *online* reports   

**Figure 6:**
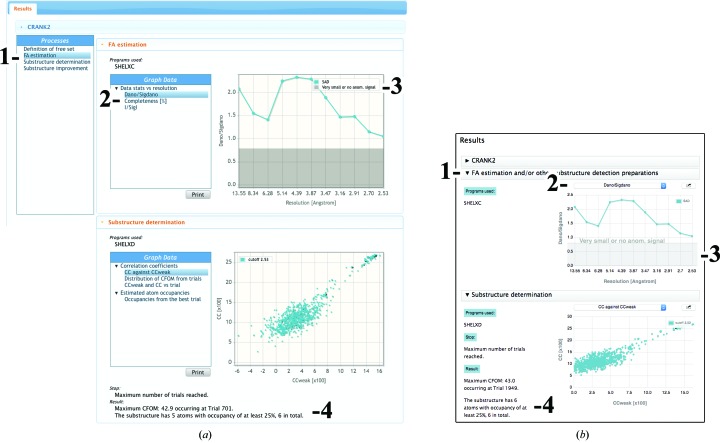
Correspondence between the graphical elements of *CCP*4 *online* (*a*) and *CCP*4*i*2 (*b*) reports. Although the underlying data are strictly the same, a different layout is imposed on *JSrview* reports for reasons of consistency. The different processes (1) are expanded into individual tabs, with each graph being selectable from the title bar of the main graph (2). Other graphical elements include shaded areas (3), which are rendered as a separate entity and not as an additional curve, and accompanying text (4). As is the case for their *JSrview* counterparts, these reports update seamlessly in real time.

*CCP*4 *online* (https://www.ccp4.ac.uk/ccp4online) provides web-server access to a growing list of *CCP*4 programs such as *CRANK*2, *PISA* and *MrBUMP*. *JSrview* (http://www.ccp4.ac.uk/dist/checkout/jsrview) is a function call-driven, server-side *CCP*4 framework that provides a report mechanism for *CCP*4 *online*. As a matter of convenience for developers, a translation mechanism between *JSrview* and *CCP*4*i*2 reports has been included. The module, CCP4­RvapiParser, offers a class which, by means of inheritance from the standard Report class, offers all of the basic *CCP*4*i*2 reporting functionality while performing the conversion automatically upon detecting changes on a separate XML file (i2.xml). This file, which is produced by *JSrview* every time the report should be updated, comprises all graph data points, presentation details (for example line colour and thickness) and accompanying text strictly required for the report (Fig. 6 ▸).

## 3. User-interface utilities   

### 3.1. Export and import of projects   

This utility enables users to transfer a project or selected jobs between computers. The tools are also used to automatically transfer a limited amount of data necessary to run a job on a server (see below). The basic mechanism could also support the transfer of information to or from electronic notebook systems, although some software-specific functionality would be needed to create a user-friendly automatic communication mechanism.

The contents of the database, either for an entire project or for selected jobs, can be written to an XML file. To export a project, the contents of the project directory (for example data files) are bundled into a compressed archive file along with an XML representation of the corresponding database entries. Since the project directory is organized with the files for one job in one job directory, it is straightforward to select only the relevant job directories for export. The compressed file can be reimported into another *CCP*4*i*2 database; this is performed with careful checking of consistency between the database and the unpacked project directory and using the database data item UUIDs to ensure that data are neither duplicated nor inappropriately overwritten.

### 3.2. Running jobs on a server   

*CCP*4*i*2 provides mechanisms to run jobs that require heavy computing resources on a server machine. The server machine must have a suitable version of the *CCP*4 suite installed. The client *CCP*4*i*2 must be configured by a system administrator or a knowledgeable user to choose the server machines, the communication mechanism, the *CCP*4 installation directory and the temporary filesystem space on the server machine. After configuration the user need only choose a server, if more than one is provided, and, depending on the communication mechanism, provide a login name and password to run a job on the server.

*CCP*4*i*2 currently supports three possible communication mechanisms:

(i) *via* ssh (implemented *via* the Paramiko library; http://www.paramiko.org/),
(ii) *via* queuing software that supports qsub standard commands (tested for the Sun Grid Engine; http://www.oracle.com/technetwork/oem/grid-engine-166852.html),
(iii) *via* a custom system. This is necessary when using a server with its own customized, potentially web-based, interface. There is documentation and examples for the user to implement their own communication system. An example of this has been implemented for the SCARF server (http://portal.scarf.rl.ac.uk) run by the Science and Technology Facilities Council in the UK.

For all three mechanisms a fragment of the project is exported, as described above, and copied to the server machine, where it is unpacked into a temporary project directory and database. Running the job on the server updates the contents of the temporary project directory and database and, when the job finishes, these are exported to a compressed file that is returned to the client machine and reintegrated into the user’s project directory and database. This mechanism requires minimal communication between client and server, but the user is informed of progress by updates to the ‘running’ report which is implemented by the client periodically retrieving the program.xml data file from the server.

### 3.3. Organizing and searching jobs and projects   

Given the large number of structure-solution projects undertaken by most crystallographers, organization of projects and data is vital. The *CCP*4*i*2 user interface provides several ways for crystallographers to organize their work and to label and annotate their files, jobs and projects. This additional information may be helpful when reviewing the work in the future.

The ‘Projects manager’ window in *CCP*4*i*2 provides tools to organize and export or import projects. The most useful organizational tool is that projects can be grouped into folders and the folders can be nested. There is additionally the option to tag and provide a description of the project. There are project-search tools based on project name, description, tags or the date the project was in use. It is also possible to search for projects that have used a user-specified file.

*CCP*4*i*2 has tools to search the jobs in a project based on simple properties such as the task name, text in the annotation or comments and when the job was run. There are also more sophisticated searches to find jobs that ran with given values of a particular control parameter and to show the progress of a given data object through a project. For the latter search the user can select any data object, input or output for a given job, and all jobs that used these data, either before or after the selected job, will be highlighted in the job list. For some types of data, particularly the model coordinates, the data values will be updated in many jobs so that the output data object (*i.e.* the data file) is different from the input data object, but the search procedure can track these changes. The uses of a data object may branch, for example, to produce several possible ‘final’ model coordinates. The interface enables the user to highlight either the jobs in a selected branch or jobs in all branches.

### 3.4. Viewing old *CCP*4*i* projects   

The important conceptual differences between *CCP*4*i* and *CCP*4*i*2 make it impossible to work with both interfaces interchangeably: for example, *CCP*4*i* allowed the rerunning of jobs and therefore the overwriting of files, so that the tracking of file provenance within the older system is not reliable. However, a mechanism has been implemented to view old *CCP*4*i* projects within the new interface and to select and import files.

*CCP*4*i*2 can read the database and project files from the *CCP*4*i* user interface and display the projects, jobs and files in the style of the job list of the new interface. Users can view log files and data files and can drag and drop the files listed in the job list from *CCP*4*i* projects into jobs in *CCP*4*i*2 projects.

## 4. The tasks   

**Figure 7:**
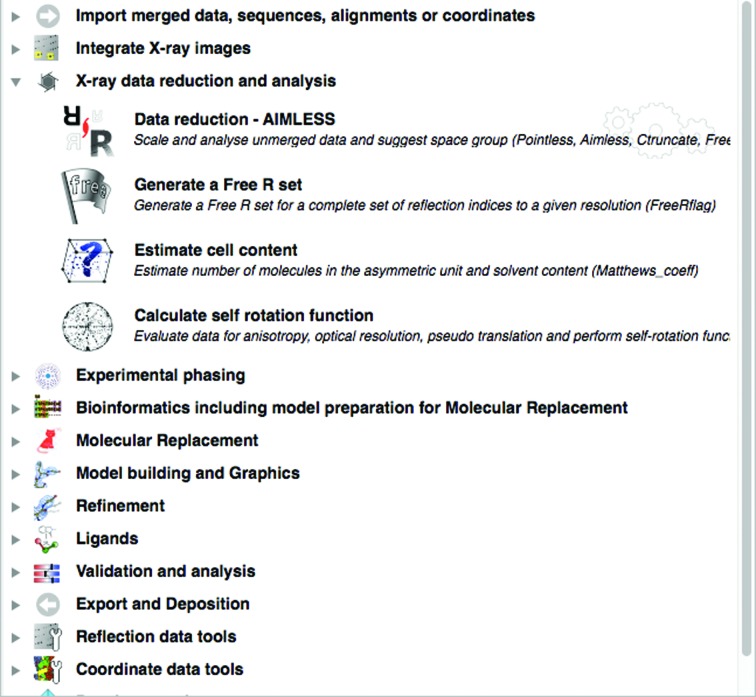
The task menu with the folder for the ‘X-ray data reduction and analysis’ module open showing the tasks in that module.

*CCP*4*i*2 provides task interfaces to the main macromolecular crystallographic structure-solution programs provided by the *CCP*4 suite. The tasks which use these programs are organized into various sections in the task list (Fig. 7 ▸). The task list is arranged to guide the user through the process of solving a crystal structure, starting from data processing and finishing with deposition. The major tasks in each section of the task list are described below.

### 4.1. Integrate X-ray images: *xia*2   

The expert system *xia*2 (Winter *et al.*, 2013 ▸) provides fully automated data processing from diffraction images to scaled and merged data. As a decision-making pipeline it uses other software to perform discrete tasks such as indexing, integration and scaling. The quality of the results is assessed at each stage, informing decisions in a dynamic manner. The capability of the software is now such that it can stand in for an expert crystallographer even in challenging cases. It is of particular use when driving command-line programs that do not have their own built-in graphical interfaces and can be intimidating or tedious to operate for many users. Although *xia*2 is itself primarily a command-line program, it has a structured interface for optional parameters using the Phil syntax of *cctbx* (http://cctbx.sourceforge.net/libtbx_phil.html). This interface is rich enough to describe the basic components of a GUI, including parameter types, tooltips, help strings and expert levels. It is possible to map most elements of the Phil interface onto a def file. For this reason, the *xia*2 interfaces in *CCP*4*i*2 differ from most other interfaces in that we choose not to use *defEd* or boilerplate code templating to design the GUI, but rather we autogenerate them from the Phil definitions. In future this may be a convenient mechanism for creating GUIs for other *cctbx*-based software.

There are two interfaces to *xia*2 automated processing in *CCP*4*i*2, one specific for the *DIALS* package (Winter *et al.*, 2018 ▸), which is included in *CCP*4, and another for *XDS* (Kabsch, 2010 ▸), if the user has it installed. Much code is shared between the two interfaces and the split into separate tasks is performed for the convenience of the user. Many of the optional parameters are specific for either *DIALS* or *XDS* and in each task irrelevant parameters can be hidden. In either case, *xia*2 uses *CCP*4 software to calculate merging statistics, which allows the *xia*2 tasks to reuse the data-reduction task report code, ensuring consistency with the data-reduction pipeline described below.

*CCP*4*i*2 also integrates the *iMosflm* GUI to *MOSFLM* (Powell *et al.*, 2017 ▸) and records the activities of this GUI in the *CCP*4*i*2 database.

### 4.2. X-ray data reduction and analysis: *AIMLESS*   

**Figure 8:**
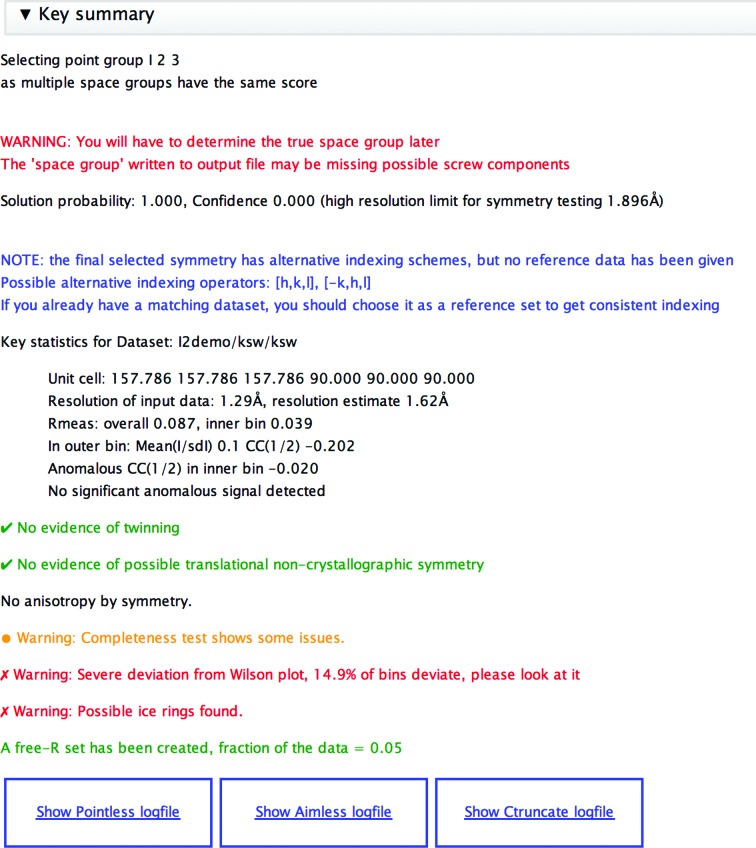
The main summary report from the Data Reduction pipeline (also used as part of the *xia*2 task). This contains the principal results and warnings of potential problems.

**Figure 9:**
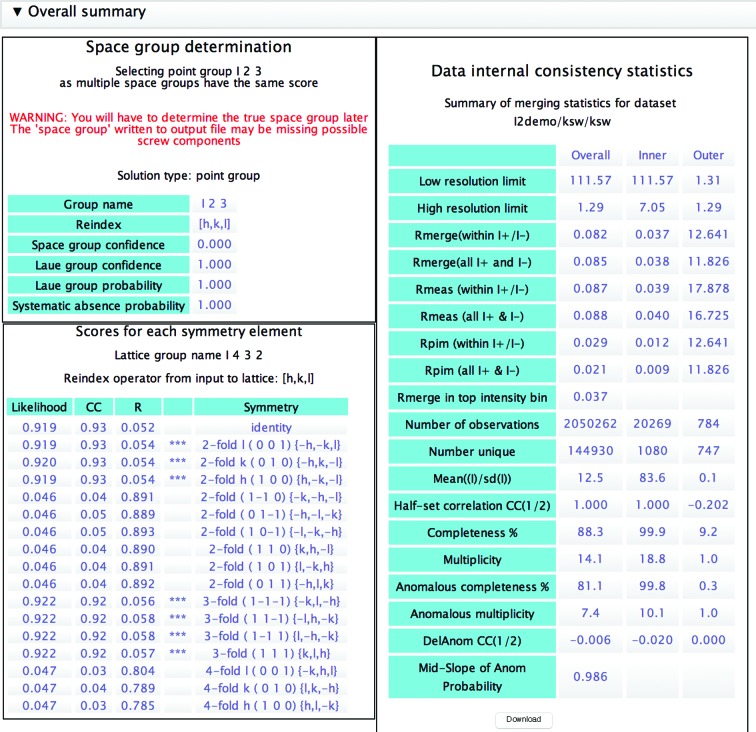
Overall summary from the Data Reduction task, including a ‘Table 1’ which can be downloaded as a CSV file for inclusion in other documents.

This is the principal task for data processing following the integration of intensities from images. As input it takes one or more unmerged and (usually) unscaled data sets from integration tasks, either within *CCP*4*i*2 (*xia*2 or *MOSFLM*) or from external sources. Even if an automated pipeline such as *xia*2 has scaled and merged the data, it may be worth re­running the data-reduction step to perform a more careful analysis of the results. The pipeline runs a series of programs, producing two output data objects for structure solution and refinement: a merged set of observed intensities (and their corresponding amplitudes) and a free-*R* set of reflections. In turn, these programs are *POINTLESS* (Evans, 2011 ▸), which determines the point group and, if possible, the space group; *AIMLESS* (Evans & Murshudov, 2013 ▸) to scale and merge the data; *CTRUNCATE* to generate amplitudes from intensities; and *FREERFLAG* to generate or extend a free-*R* set of reflections for refinement. The pipeline generates an extensive report, highlighting any noteworthy issues (Fig. 8 ▸) such as alternative indexing (in which case a reference data set may be provided to obtain consistent indexing). The report is organized with overall summaries at the top (Fig. 9 ▸), and offers the possibility of drilling down to more detailed graphs and tables. After examination of the report, it is common to rerun the task, changing the resolution limits, omitting some parts of the data or merging multiple isomorphous data sets.

### 4.3. Experimental phasing: the *CRANK*2 and *SHELXC*/*D*/*E* pipelines   

*CRANK*2 (Skubák & Pannu, 2013 ▸) and *SHELXC*/*D*/*E* (Sheldrick, 2010 ▸) are pipelines for automated structure solution from SAD, MAD or SIRAS experimental data. The *SHELXC*/*D*/*E* pipeline is implemented as a subset of *CRANK*2, calling *SHELXC*, *SHELXD* and *SHELXE* (Sheldrick, 2010 ▸) followed by *Buccaneer* and *REFMAC*5 *via* program wrappers. In this way, most of the *CCP*4*i*2 code for the *CRANK*2 and *SHELX* pipelines is shared and thus provides identical input and output to the user. The *CRANK*2 interface also supports the MR-SAD experiment – (re)building of an input partial model, typically found by molecular replacement – using the SAD data with the powerful ‘combined’ algorithm (Skubák & Pannu, 2013 ▸). *CRANK*2 has wrappers for a variety of programs, including *SHELXC*, *SHELXD*, *SHELXE*, *PRASA* (Skubák, 2018 ▸), *Parrot* (Cowtan, 2010 ▸), *SOLOMON* (Abrahams & Leslie, 1996 ▸), *ARP*/*wARP* (Langer *et al.*, 2008 ▸), *Buccaneer* (Cowtan, 2006 ▸) and *REFMAC*5 (Murshudov *et al.*, 2011 ▸).

The pipeline is composed of several steps: from determination of the anomalous substructure, through phasing, hand determination and phase improvement, to model building. Each of the steps behaves as a separate *CCP*4*i*2 wrapper, so it is possible to clone any of the steps and rerun it with modified parameters. Furthermore, the pipeline can be customized to start and stop at any step: for example, the pipeline can be started from substructure phasing by inputting a known substructure, or can be stopped immediately after a substructure has been determined. A *CCP*4*i*2 running report indicates the progress of each step and, for many steps, a button is provided to stop at the end of the current cycle and proceed to the next step, should the user find the current performance satisfactory (for example, to proceed to phasing if the user believes that the substructure has been adequately determined).

The minimal required user input consists of the anomalous data set (or multiple data sets in the case of MAD or SIRAS experiments), sequence description and anomalous heavy-atom type. The partial model, typically from MR, needs to be inputted for MR-SAD. While the compulsory input is sufficient for many data sets, additional options are provided to tackle data sets that are difficult to solve or to optimize the structure-solution process. The default values for all of the options are input-dependent and are dynamically generated and subsequently displayed in the graphical user interface as soon as the minimal user’s input is provided. Since the GUI calls *CRANK*2 to generate the defaults, a typical problem of duplication of interface and the underlying program defaults is prevented.

There are also interfaces to phasing using *Phaser* and to density modification with *ACORN* and *Parrot*.

### 4.4. Model preparation for molecular replacement: *CCP*4*mg* and *MrBUMP*   

*MrBUMP* (Keegan & Winn, 2008 ▸) is an automated scheme for molecular replacement with its own *CCP*4*i*2 interface. Given a target sequence and measured reflections, it will search for homologous structures, create a set of suitable search models from the template structures, perform molecular replacement and test the solutions with some rounds of restrained refinement. *MrBUMP* has been integrated into the *CCP*4*mg* (McNicholas *et al.*, 2011 ▸) molecular-graphics program for interactive model preparation, and this module can be called directly from *CCP*4*i*2. A fuller description of this task is given in the article on *MrBUMP* in these proceedings (Keegan *et al.*, 2018 ▸).

*CCP*4*i*2 also has task interfaces to run the molecular-replacement model-preparation tools *CHAINSAW* (Stein, 2008 ▸), *Sculptor* (Bunkóczi & Read, 2011 ▸) and *Ensembler* (part of the *Phaser* suite; McCoy *et al.*, 2007 ▸), and the sequence-alignment tool *ClustalW* (Larkin *et al.*, 2007 ▸).

### 4.5. Molecular replacement   

*CCP*4*i*2 provides task interfaces to MR using the *MrBUMP* pipeline, *MOLREP* (Vagin & Teplyakov, 2010 ▸) and *Phaser*. There are two versions of the *Phaser-MR* interface: a basic mode for a single search model and an expert mode for more complex models and crystal composition that provides access to more advanced keyword options.

### 4.6. Refinement: *REFMAC*5   

The Refinement pipeline is the main task for performing model refinement using *REFMAC*5 (Murshudov *et al.*, 2011 ▸), optionally using additional restraints from *ProSMART* (Nicholls *et al.*, 2014 ▸). Model coordinates, reflection data and, usually, a free-*R* set are provided as the main inputs. Optional additional inputs include experimental phase information, TLS coefficients, ligand dictionaries and reference models. TLS coefficients, which describe a concerted screw motion of rigid bodies (Winn *et al.*, 2003 ▸), can be imported and/or edited using the corresponding utility also found in the ‘Refinement’ menu. Bespoke ligand dictionaries (CIF files) can be generated using the ‘Make Ligand’ task in the ‘Ligands’ menu. If a reference homologous model is provided then *Pro­SMART* will be executed by the pipeline to generate external restraints. These restraints will then be automatically used by *REFMAC*5 during refinement: this option may be suitable at lower resolutions (Nicholls *et al.*, 2012 ▸).

The main refinement options include the number of cycles, the geometry weight, whether or not the crystal is believed to be twinned and whether riding H atoms are to be generated and used. By default, the geometry weight is automatically adjusted during refinement in order to ensure a reasonable balance between prior information (geometry) and observed data. If twinning is assumed, refinement can be performed against structure-factor amplitudes or intensities, depending on the nature of the main input data. Additional options pertain to model parameterization (*B* factors, TLS groups), prior information (NCS restraints, jelly-body restraints) and the scaling method (solvent model). The ability to perform anisotropic regularized map sharpening when calculating electron-density maps is also provided, noting that this option does not affect refinement of the model: it just modifies the output maps.

Compared with the equivalent task in *CCP*4*i*, the Refinement interface in *CCP*4*i*2 is relatively minimalist so as to avoid presenting the user with an overwhelming number of modes and options. Only the options that are most commonly required by a standard user are presented in the interface, although additional keywords can be provided in order to enable expert users to tweak advanced options and parameters should they wish. Note that the pipeline is designed specifically for full-model restrained refinement. For clarity of application, some of the other functional modes of *REFMAC*5 (un­restrained refinement, rigid-body refinement and structure idealization) are excluded from the interface owing to being outside the scope and being deemed to be of less practical use during the course of the modern crystallographic structure-solution process. Note also that whilst it was previously common for rigid-body refinement to be performed straight after molecular replacement, it is now more typical to execute a large number of cycles of jelly-body restrained refinement.

When the job is running, the results page is iteratively updated in order to provide the user with feedback regarding the current status of refinement. Global refinement statistics, the current geometry weight and graphs corresponding to the per-cycle evolution of *R* factors and r.m.s.(bonds) are updated each refinement cycle, expediting manual assessment regarding job quality (monitoring refinement convergence, excessive overfitting *etc.*), which, in the case of a suboptimal result, may lead to the user killing the job, cloning and re-running it using different parameter values or options. A successfully executed job can be immediately followed by further refinement using the Refinement pipeline, manual model building using *Coot* or autobuilding using *Buccaneer*.

### 4.7. Model building and graphics   

Tasks are provided for both manual and automated model building. Automated model building is implemented both within experimental phasing pipelines (in particular the *CRANK*2 and *SHELX* tasks) and in standalone model-building tasks. Tasks are implemented for protein model building using either the *Buccaneer* or *ARP*/*wARP* software (Cowtan, 2006 ▸; Langer *et al.*, 2008 ▸).

#### 4.7.1. Manual model building with *Coot*   

The *Coot* task starts the *Coot* molecular-graphics program (Emsley *et al.*, 2010 ▸) with data from the *CCP*4*i*2 project. The task input allows arbitrary numbers of coordinate sets, density and difference density maps to be loaded into the *Coot* session. Additionally, a ligand dictionary may be specified. *CCP*4*i*2 can also generate scripts to automate some of the tasks in the *Coot* session, and such scripts may be loaded through this task interface. During a *CCP*4*i*2 *Coot* session, the menus of *Coot* are modified to allow the saving of coordinate sets into the *CCP*4*i*2 database, with these coordinate becoming available to subsequent *CCP*4*i*2 tasks when the *Coot* session is exited. Similarly, ligand dictionaries generated by tools with *Coot* are identified when the *Coot* session ends, and are also made available for use in subsequent jobs.

A further *CCP*4*i*2 extension menu allows the user access to all coordinate, map and dictionary data objects that are known to the user’s database, allowing rapid comparison of maps and models from different stages of the refinement procedure. *Coot* launched from *CCP*4*i*2 automatically includes various key bindings, which were developed to allow easy ‘one-key’ access to various heavily used functions of *Coot* during manual model building.

#### 4.7.2. Scripted model building with *Coot*   

In this task *Coot* is run without opening the *Coot* GUI. A *Coot* script file is necessary to define the operations that *Coot* will perform. The model, map and dictionary inputs are the same as in the ‘Manual *Coot*’ task, but there are more options provided by *CCP*4*i*2 (fill partial residues, fit protein, stepped refinement with or without Ramachandran restraints and iterative morph fit) and there is the option to enter a script manually.

There is also a separate task ‘Find Waters’, which takes a map and a model and runs *Coot* in windowless mode to find water molecules.

#### 4.7.3. Autobuild protein   

This tool supersedes the *Buccaneer* pipeline in *CCP*4*i*, introducing a range of new possibilities provided by the scripted use of *Coot*. It consists of iterations of *Buccaneer* (Cowtan, 2006 ▸), *Coot* and *REFMAC*5, with the optional use of *ProSMART*. The main inputs vary according to the provenance of the phases being used. If these were produced in an MR job, the MR solution must be supplied in order to have *Buccaneer* take advantage of it: placing the new model and naming chains after it and, optionally, deriving the first set of coordinates from either the complete MR solution or an edited version of it, known as seeding mode. If no phase estimates are provided, the MR solution is refined in *REFMAC*5 in order to produce them. If the initial phases come from experimental methods they must be supplied explicitly, and in this case the user is provided with an option to specify a model *R*-factor threshold below which experimental phases are no longer used in iterative model building and phase combination.

As they are very dependent on map interpretability, real-space refinement options are only available after the *R* factors go below a certain threshold: this is also configurable, although a reasonable estimate of 0.35 is provided by default. Backrub rotamers are used by default within *Coot* rounds.

Except in the first iteration, map coefficients and the refined autobuilt model produced by *REFMAC*5 in the previous iteration are supplied as input to *Buccaneer*. The input model is then filtered (badly fitting regions are removed), extended and refined again, with most problems being driven to convergence in ∼5 iterations (MR phases) or ∼15 iterations (experimental phases).

The *CCP*4*mg* task starts the *CCP*4*mg* molecular-graphics program (Mc­Nicholas *et al.*, 2011 ▸) with data from the *CCP*4*i*2 project. The task input allows arbitrary numbers of coordinate sets, density and difference density maps, and sequences to be loaded into the *CCP*4*mg* session.

### 4.8. Ligands   

To refine a structure containing novel ligands, the crystallographer needs provisional starting coordinates for the ligand and a geometry dictionary. These can be created within *CCP*4*i*2 using *AceDRG* (Long *et al.*, 2017 ▸) *via* the ‘Make Ligand’ task. This task also allows a molecular sketch of the ligand to be provided as a starting point, using the two-dimensional ligand-sketching capability provided by the *Lidia* program (part of *Coot*; Emsley *et al.*, 2010 ▸).

As an illustration of the power of the array of crystallographic functionality that has been wrapped for use in *CCP*4*i*2 as described above, we have also developed a ligand pipeline that spans the entire workflow from data reduction to ligand building and automatic ligand placement to cater for the case of investigating fragment and/or drug binding to a well characterized crystal system. The ligand pipeline embeds (i) the ‘Make Ligand’ task, (ii) the data-reduction pipeline, (iii) rigid-body refinement within *Phaser* and (iv) nongraphical scripted running of *Coot* to perform the actual ligand fitting. As an alternative to rigid-body fitting within *Phaser*, the user can select to use the *DIMPLE* pipeline (Wojdyr *et al.*, 2013 ▸) as an engine for rigid-body refinement.

To facilitate the use of this ligand pipeline on the tens of data sets that may be collected in a single synchrotron trip, we have also developed a meta task that (i) investigates the directory hierarchy of files returned from a Diamond Light Source synchrotron trip, (ii) generates a *CCP*4*i*2 project for each data set identified and (iii) launches the ligand pipeline in each project using a user-specified SMILES string to define the ligand associated with each data set and a common starting model.

Taken together, these tools allow a user to apply best-of-breed tools uniformly to tens of data sets in a single task, for which the total setup time may be only a few minutes. In a multiprocessing environment, comprehensive analysis can be completed in less than an hour. The outputs of this approach can also trivially be provided to *PanDDA* (Collins *et al.*, 2017 ▸) to identify low-occupancy binding events.

### 4.9. Validation and analysis   

#### 4.9.1. Validation of carbohydrate structures: *Privateer*   

**Figure 10:**
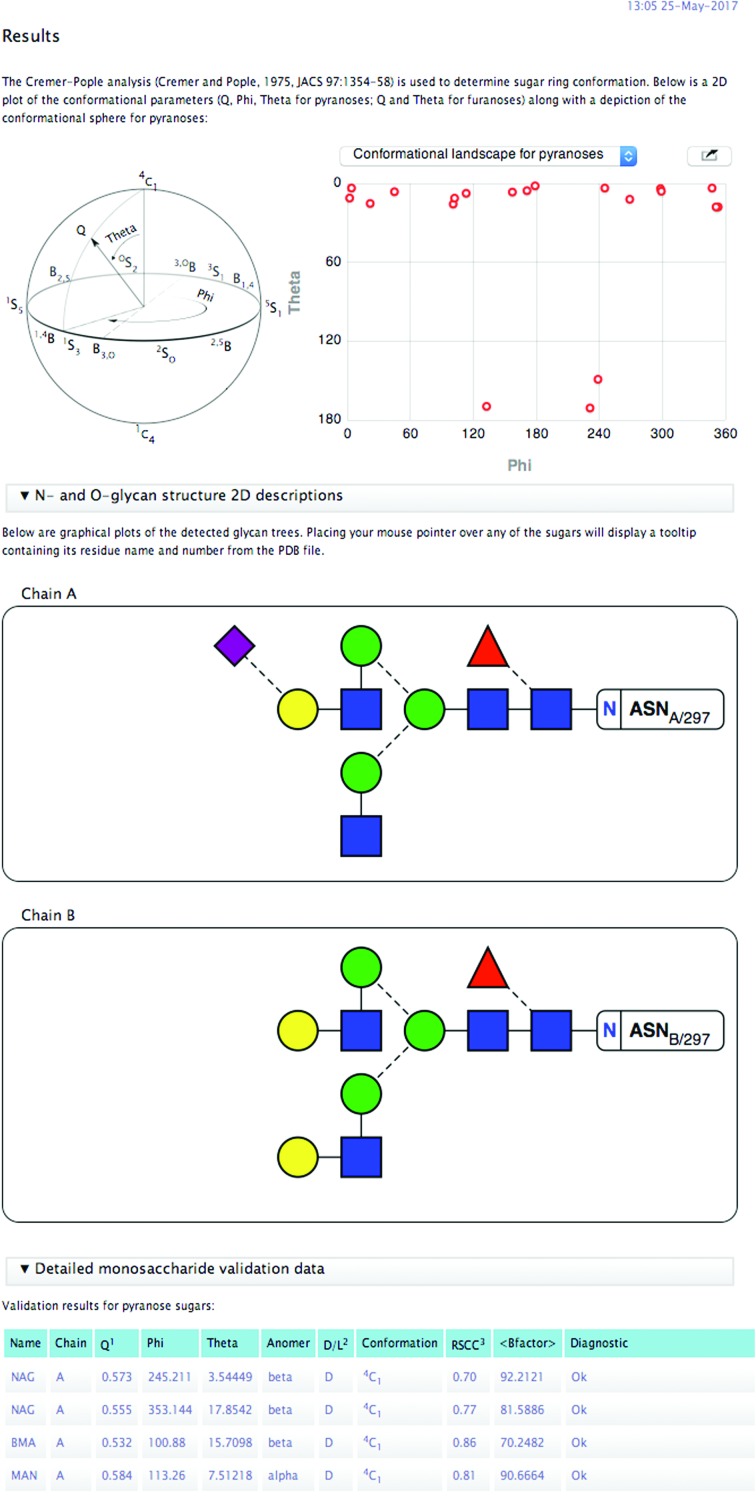
Results page after running *Privateer* on PDB entry 4byh. The report includes a conformational analysis of the monosaccharides automatically found in the supplied structure, plus additional graphs of real-space correlation coefficient *versus* *B* factor and others. Whenever any type of glycosylation is found, the report will also include two-dimensional vector diagrams of the trees, which are generated according to the notation in the third edition of *Essentials of Glycobiology* (Varki *et al.*, 2015 ▸).

The *Privateer* software was first released by *CCP*4 in 2015 (Agirre, Iglesias-Fernández *et al.*, 2015 ▸) as a tool to aid in the refinement, validation and graphical analysis of glycans. It is able to perform conformational analysis, density correlation against OMIT maps and analysis of link anomericity and torsions, and presents the results both in tabulated form and as vector graphics (SVG; see Fig. 10 ▸).

The graphical frontend bundled with *CCP*4*i*2 allows the correction of conformational anomalies (Agirre, Davies *et al.*, 2015 ▸) using the dictionaries that *Privateer* produces. These will appear as input in any subsequent *Coot* or *REFMAC*5 job. Additionally, *Coot* jobs will receive a Python script that will guide the user through the detected issues, activate torsion restraints and colour the OMIT maps.

#### 4.9.2. Analyse fit between model and density   

The density-correlation tool *EDSTATS* (Tickle, 2012 ▸) has been bundled in a completely different way to how it was in *CCP*4*i*: instead of producing a comprehensive frontend for the program, a pipeline covering data conversion and analysis has been developed, making the analysis of the results more straightforward.

As map coefficients (**F**, φ) are the preferred representation for maps within *CCP*4*i*2, whereas *EDSTATS* requires oversampled map files, a pre-processing step using *CFFT* has been added. This generates the map files in the required format transparently to the user. Also, within the interface a set of configurable thresholds can be set for the different accuracy and precision metrics, separated by protein main chain and side chain. The outliers found using these criteria are listed in a Python script that can be used in a subsequent *Coot* job, giving the user the possibility to track and fix them up quickly. Isolated main-chain outliers can typically be improved by flipping the peptide, while fixing side-chain outliers will probably involve a rotamer search.

## 5. Summary and prospects   

*CCP*4*i*2 now provides a computing environment in which productive crystallography can be accomplished and an effective record of the structure-determination process can be retained. The current focus of the development team is to consolidate and extend the existing functionality, for which user feedback would be gratefully received. Other planned developments include enabling group access to *CCP*4*i*2 projects by introducing a client–server database-management system to be available as well as the current onboard SQLite system and access to centralized computation servers from *CCP*4*i*2. We expect that over the lifetime of *CCP*4*i*2 the structure-solution process will become more automated, and the system provides a sound basis for automation while still enabling crystallo­graphers to view the details of the process and intervene when they need to.

For program and workflow developers, *CCP*4*i*2 provides a framework in which aspects of pipelining, data tracking and graphical report presentation are provided with a relatively low overhead for task implementers. The development team will welcome prospective developers and support them in making their software accessible *via* *CCP*4*i*2. The modular design of wrappers and incremental building of pipelines will enable increasing automation, but by providing graphical tools for users to review and control tasks we can avoid the structure-solution process becoming a black box. *CCP*4*i*2 is well positioned to support users and developers through the next period of increased throughput and output of macromolecular crystallography and related disciplines.

## 6. Availability   

*CCP*4*i*2 can be obtained from http://www.ccp4.ac.uk/download as part of the *CCP*4 suite of programs.

## Figures and Tables

**Table 1:** Third-party Python libraries bundled in ccp4-python and used in *CCP*4*i*2

Python library	Function	URL
lxml	Handling XML files	http://lxml.de
numpy	Scientific computing	http://www.numpy.org
matplotlib	Two-dimensional graph plotting	https://matplotlib.org
paramiko	Inter-machine communication	http://www.paramiko.org/
psutil	Access operating-system utilities	http://pypi.python.org/pypi/psutil

**Table 2:** The key tables in the *CCP*4*i*2 database

Main database table	Represents	Key data
Users	*CCP*4*i*2 user	User name
Projects	The structure-solution project	User ID, project name, directory, parent project
Jobs	A job or sub-job	Project ID, parent job ID, task name, status, job title
Files	Files imported or created in the project	Job ID, file path, annotation, file type, subtype, file content
File uses	File input to a job	File ID, job ID
Import files	Source of a file that was imported to the project	File ID, source file path, annotation
Job key values	Key progress data for job	Job ID, data type, data value
Comments	User comment on job	User ID, job ID, text
Project comments	User comment on project	User ID, project ID, text
